# The role of non-HDL cholesterol and atherogenic indices in predicting poor glycemic control among type 2 diabetic patients in Basrah, Iraq

**DOI:** 10.5339/qmj.2022.32

**Published:** 2022-08-02

**Authors:** Jasim N Al-Asadi, Lamia M Al-Naama

**Affiliations:** College of Medicine, University of Basrah, Basrah, Iraq E-mail: jnk5511@yahoo.com

**Keywords:** Dyslipidemia, diabetes mellitus, glycemic control, glycosylated hemoglobin, lipid ratios

## Abstract

Background: Dyslipidaemia is frequently associated with type 2 diabetes mellitus and it is the major contributor to cardiovascular diseases among type 2 diabetic patients. Despite the fact that several researches have proven the association between glycemic control and dylipidemia in type 2 diabetic patients, the results are rather varied.

Objectives: The aim of the study is to investigate the clinical relevance of lipid profile as predictive biochemical model for glycemic control in type 2 diabetic patients.

Methods: A cross-sectional study including 329 type 2 diabetic patients was done in Al-Sadr Teaching Hospital, Basrah, Iraq. Brief history, clinical examination, and investigations including fasting plasma glucose, lipid profile, and glycosylated hemoglobin were done. HbA1c >7% was considered as poor glycemic control. Receiver operator characteristics (ROC) analysis and logistic regression analysis were used to evaluate the association between lipid profile and HbA1c level.

Results: Out of 329 diabetic patients, 278 (84.5%) showed poor glycemic control. The univariate analysis showed a significant association between lipid parameters and poor glycemic control. ROC and logistic regression analyses found that TC/HDL (OR: 4.94; 95% CI: 2.35–10.41; P < 0.001) and LDL/HDL (OR: 4.63; 95% CI: 1.96–10.98; P < 0.001) were the only significant independent predictors of glycemic control, while non-HDL cholesterol was a weak predictor of glycemic control despite its significant association (P = 0.02).

Conclusion: LDL/HDL and TC/HDL ratios reveal promising indicators for predicting glycemic control in type 2 diabetic patients.

## Introduction

Diabetes mellitus (DM) is a global health problem. According to the International Diabetic Federation, the global prevalence of DM was 10.5% in 2021 and is projected to reach 11.3% in 2030 and 12.2% in 2045. DM or its complications killed an estimated 6.7 million people aged 20–79 years in 2021, accounting for 12.2% of all-cause mortality. Adults with DM comprise 80.6% of the population in low- and middle-income countries and are expected to comprise 84.9% of the population in 2045. In 2021, the estimated prevalence of DM in Iraq was 10.7% and it is expected to rise to 11.6% in 2030 and to 12.2% in 2045^1^. The overall adjusted prevalence of DM in Kuwait is 19.1%^2^, 13.6% in Turkey^3^, and 15.3% in Khuzestan, Iran^4^.

The rising prevalence of DM^1^ indicates an increase in diabetes-related complications in the future. The potential concern for health professionals will be to provide appropriate treatment for DM patients to attain the best glycemic control possible^5,6^. Because of the close relationship between glycemic control and the lipid profile, both conditions must be carefully monitored to avoid diabetes-related macrovascular and microvascular complications^7,8^.

Despite its limitation in patients with hemoglobinopathies^9^, glycosylated hemoglobin (HbA1c) is the gold standard for determining glycemic control in DM patients^10^. A high and variable HbA1c level is a reliable risk factor for cardiovascular disorders and all-cause mortality in patients with DM^11^.

Low density lipoprotein cholesterol (LDL-C) is an important focus in the treatment of lipid profiles in patients with coronary artery disease (CAD) or a CAD risk factor counterpart, such as diabetes, according to the National Cholesterol Education Program Treatment for Adults Panel III (NCEP ATP III)^12^ and the European Society of Cardiology^13^. The level of LDL-C in patients with DM may not be elevated, so the cardiovascular risk is not specifically known^14^. As a result, the recent NCEP ATP III guidelines^15^ and several other studies^16,17^ emphasized non-high density lipoprotein (non-HDL) cholesterol as a useful predictor of cardiovascular risk. This is particularly applicable if the triglycerides level is high. Non-HDL cholesterol is calculated as the difference between total and HDL cholesterol and thus reflects the sum of cholesterol carried on potentially prothrombotic apolipoprotein B-containing particles^16^. It is a practical, reliable, and inexpensive measure, and is accepted as a surrogate for apolipoprotein B (apo B) in routine clinical practice when apo B is not available^17^. Apolipoproteins include apoA, apoB, apoC, apoD, apoE, and apoM. Apolipoproteins are key components of plasma lipoproteins that transport lipids and maintain lipoprotein structure. DM is characterized by complex apo metabolic abnormalities, such as high plasma concentrations of apoB, apoC II, apoC III, apoE, and low plasma apoA I and apoM concentrations, which are associated with dyslipidemia and its related complications^18^.

Several studies have revealed that diabetic patients have abnormal levels of lipids and there is a significant relationship between the lipid profile and the HbA1c level and vice versa^19,20^ Growing evidence indicates an association between non-HDL cholesterol and glycemic control in diabetic patients^21^.

The prevalence of DM, which is associated with an increase in complications and mortality, is rapidly increasing worldwide and at the national level^1^. Uncontrolled DM is highly prevalent in Iraq with a rate of 86.2%^22^. Several studies have stressed the role of dyslipidemia in glycemic control in DM patients, yet the usefulness of certain lipid parameters is inconsistent in some cases^23,24^. Considering the high cost of measuring various plasma lipid markers, such as apolipoprotiens, in resource-limited settings, the use of simply calculated and low-cost lipid ratios would greatly aid in assessment of glycemic control. Thus, in addition to the lack of studies in Iraq that have investigated the relationship between non-HDL cholesterol and glycemic control, this study was carried out to identify the role of non-HDL cholesterol and the lipid ratios “atherogenic indices” in predicting such control in patients with type 2 DM in Basrah, Iraq.

## Materials and methods

### Study design and participants

This cross-sectional study was performed at the outpatient department of Al-Sader Teaching Hospital, Basrah, Iraq, and included patients with known type 2 DM. Patients with diseases that impair glucose control, such as chronic hepatic or thyroid disorders, pregnant women, and patients with known hemoglobinopathies were excluded from the study. Patients were assigned to one of two groups based on their glycated hemoglobin value: group 1 included patients with good glycemic control (HbA1c ≤ 7%), and group 2 included patients with poor glycemic control (HbA1c>7%)^7^.

### Sample size and sampling

The sample size was determined with a formula^25^. A minimum sample size of 316 was calculated using a reliability coefficient (Z score) of 2.576 at a 99% confidence interval, a margin of error of 5%, and an uncontrolled diabetes prevalence rate of 86.2%^22^. After accounting for a 5% non-response rate, the overall sample size was 332. Three patients had incomplete biochemical data, so the remaining 329 (99.1%) comprised the study population. A simple random sampling was used to choose the required number of participants from those who consecutively attended the outpatient clinic of the hospital each day the researcher visited the clinic until the targeted number was achieved.

### Data collection

Sociodemographic and clinical information was obtained using a questionnaire interview. Height and weight were measured, and the body mass index (BMI) was calculated. Blood pressure was measured using a mercury sphygmomanometer while the patient was in a sitting position after a 5 min rest. The average of two readings 5 min apart was the result.

### Biochemical measurements

Blood samples were collected after a 12-hour overnight fast. An aliquot of blood (2 ml) was collected in EDTA anticoagulant for the HbA1c assay, while the remainder was allowed to clot at room temperature, centrifuged, and the serum was used for the biochemical analysis within 1 hour. The lipid profile was determined using Roche diagnostic kits and the COBAS INTEGRA 400 plus instrument (Roche Diagnostic GmbH, Mannheim, Germany). These tests included measurements of total cholesterol (TC), high density lipoprotein cholesterol (HDL-C), low density lipoprotein cholesterol (LDL-C), and triglycerides (TGs). Non-HDL cholesterol was determined by subtracting HDL-C from TC^16^. Hemoglobin A1c was determined by ion-exchange high-performance liquid chromatography using the VARIANT ^TM^ program kit (Bio-Rad Laboratories, Hercules, CA, USA).

The lipoprotein ratios (atherogenic indices) were calculated as follows: Castelli Risk Index (CRI-I) also known as the cardiac risk ratio = TC/HDL-C and Castelli Risk Index (CRI-II) =  LDL-C/HDL-C^26^. The Atherogenic Index of Plasma (AIP) was calculated as Log10 (TG/HDL-C) ratio^27^. The blood lipid profile reference level was determined using the NCEP ATP III guidelines^28^. Hypercholesterolemia was defined as a TC level >200 mg/dl, a high LDL-C level was >100 mg/dl, a hypertriglyceridemia level was >150 mg/dl, and a low HDL-C level was < 40 mg/dl. Dyslipidemia was defined as the presence of one or more abnormal blood lipid values. The normal non-HDL cholesterol value was < 130 mg/dl. The normal values used for the lipid ratios were those used for the cardiovascular disease risk prediction; TC/HDL ratio < 5 (optimal < 3.5), LDL/HDL ratio < 3.3 (optimal < 2.5), TG/HDL ratio 3.8 (optimal < 2)^29^.

### Statistical analysis

Statistical analyses were performed using SPSS version 25.0 software (IBM Corp., Armonk, NY, USA). Categorical variables are presented as numbers and percentages and compared with the chi-square test. Continuous variables are presented as mean ± standard deviation and compared using the *t*-test.

A receiver operator characteristics (ROC) curve was used to determine the optimal cutoff values along with corresponding sensitivities and specificities of the studied lipid indices for predicting poor glycemic control in patients with DM.

A P-value < 0.05 was considered significant. Binary logistic regression analysis was performed to determine the independent variables associated with poor glycemic control.

### Ethical considerations

The Ethics Committee of the College of Medicine, University of Basrah approved this study (Project ID 030407-049-2022). Informed consent was obtained from all participants before they enrolled in the study.

## Results

This study included 329 type 2 DM patients; 169 (51.4%) were females and 160 (48.6%) were males. Among the study population, 278 patients (84.5%) had poor glycemic control. More females than males had uncontrolled DM with a highly significant difference. Glycemic control was not significantly associated with age, smoking, family history of DM, or duration of DM. Female sex, BMI, fasting blood glucose (FBG), type of treatment, and systolic and diastolic blood pressures were significantly related to the control of DM as measured by HbA1c [Table 1].

All lipid parameters and lipoprotein ratios (atherogenic indices) were significantly higher in patients with poor glycemic control, except HDL, which was inversely correlated with glycemic control [Table 2].

The ROC analysis showed that non-HDL, non-HDL/HDL, TC/HDL (CRI-I), LDL/HDL (CRI-II), TG/HDL, and the log TG/HDL (AIP) parameters exceeded TC, TG, and LDL in predicting glycemic control with an Area under the curve (AUC)>0.7 compared to an AUC < 0.7 for TC, TG, and LDL [Table 3].

The logistic regression analysis showed that the TC/HDL ratio (CRI-I) [OR: 4.94; 95% CI: 2.35–10.41; P < 0.001] and LDL/HDL (CRI-II) [OR: 4.63; 95% CI: 1.96–10.98; P < 0.001] were the most significant independent lipid parameters that predicted glycemic control with a risk of more than four times higher than those who had normal levels of these two parameters. Despite the significant association, non-HDL cholesterol was a poor predictor of glycemic control (OR: 1.01; 95% CI: 1.00–1.02; P = 0.020). In contrast, TC and TG were not significant predictors (P = 0.130 and P = 0.488 respectively). [Table 4]

## Discussion

Glycemic control in patients with DM is crucial for reducing diabetes-related complications and lowering disability and mortality^30^. The current study showed that 84.5% of the participants had poor glycemic control. A result comparable to that previously reported in Iraq (86.2%)^22^.

Studies from various countries have revealed varying rates of poor glycemic control, including Saudi Arabia (74.9%)^31^, Iran, Yazd (58.3%)^32^, and Ethiopia (72.7%)^33^. DM patients in the United States have a better glycemic control rate (about 50%) than patients in other nations^34^.

Diabetes management is challenging and not widely available in developing countries with inadequate resources^35^. The difficulties include problems with the quality and accessibility of medical care, drug supply and cost, insufficient health institute infrastructure, common use of traditional and herbal therapies, patient ignorance, and lack of information about optimal diabetes management^36^.

Multiple factors in Iraq, such as war, sectarian strife, politics, financial issues, and security concerns, have had a long-term impact on the healthcare system. As a result, primary care facilities and skills are insufficient, and healthcare delivery is mainly reliant on secondary and tertiary care. Primary care programs for early diagnosis of non-communicable diseases, including DM have been designed; however, they were unsuccessfully implemented^37^. Furthermore, patient-related factors are important in the control of DM. A study in Basrah, Iraq showed that the rate of adherence to medication regimens and lifestyle advice was unsatisfactory^38^.

Among patient demographic, anthropometric, and clinical variables, female sex, BMI, SBP, DBP, and FBG were significantly associated with glycemic control. A finding, which has been reported previously^20,39^.

Dyslipidemia may play a significant role in the pathogenesis of DM^40^. Furthermore, it is closely related to glycemic regulation^19^. Dyslipidemia in patients with DM is hypothesized to be caused by several factors, including insulin effects on liver apoprotein production, modulation of lipoprotein lipase, cholesteryl ester transfer protein reactions, and peripheral insulin actions on adipose and muscle tissue^41^.

This study showed that non-HDL cholesterol, as well as certain lipid ratios [non-HDL/HDL, TC/HDL (CRI- I), LDL/HDL (CRI- II), TG/HDL, log TG/HDL (AIP)], predicted glycemic control because they had an AUC>0.7. However, the TC/HDL and LDL/HDL ratios performed better than other lipid measures, with AUCs of 0.78 and 0.72, respectively, and cutoff values of 4.7 and 3.1, respectively, which are comparable to normal levels^29^. Additionally, these two parameters have adequate sensitivity and specificity rates. Furthermore, the logistic regression analysis showed that the TC/HDL and LDL/HDL ratios were the most significant independent risk factors of poor glycemic control with ORs of 4.94 and 4.63, respectively. Non-HDL cholesterol was a weak predictor despite its significant association (OR: 1.01; 95% CI: 1.00–1.02; P = 0.02). Our findings are compatible with those of Artha et al^7^, who reported that the TC/HDL-C and LDL-C/HDL-C ratios can be used as predictive models (AUC>0.7), with a cutoff, sensitivity, and specificity of 4.68 (77%; 52%), and 3.06 (98%; 56%) respectively. Similarly, Bal et al.^42^ detected a significant association between the TC/HDL ratio, non-HDL cholesterol, and poor glycemic control.

In contrast, Panjeta et al^43^ showed that patients with good glycemic control have lower TC/HDL ratio (CRI-1 index), LDL/HDL ratio (CRI-2 index), and AIP, compared to patients with poor glycemic control, but the difference was not significant.

The ROC analysis indicated that non-HDL is a good predictor of glycemic control with an AUC>0.7 and a cutoff value, sensitivity, and specificity of 155mg/dl, 82.4%, and 51% respectively. The univariate analysis revealed a significant association with HbA1c (P = 0.001), which agrees with the findings of Phadake et al.^21^. However, the multivariate analysis showed that the association between non-HDL and glycemic control was weak, although significant (OR, 1.01; 95% CI: 1.00–1.02; P = 0.020).

The univariate analysis revealed that all lipid parameters examined were significantly associated with poor glycemic control, which is consistent with the findings of earlier studies^44,45^ However, some studies have reported inconsistent results about the relationship between certain lipid parameters and glycemic control. Omar et al. showed that poor glycemic control is associated with a high TC level but not with TGs^23^. Another study found that HbA1c was associated with TGs and TC, rather than LDL-C and HDL-C^24^. These inconsistencies may be due in part to the relative stability of HbA1c. Although lipid profiles can change, HbA1c values remain constant through time^8^. Furthermore, other variables, such as cholesterol-lowering drugs, may affect the relationship between glycemic control and the lipid profile^46^. Statins are associated with an increase in HbA1c in DM patients when compared to placebo^47^. Metformin therapy effectively improves dyslipidemia in DM patients who are not taking statins^48^. Diabetic comorbidities, such as cardiovascular disease and nonalcoholic steatohepatitis, can also affect glycemic control and regulation of the lipid profile^40^. Similarly, a dietary intervention has a significant impact on patient FBG and lipid profiles^49^. Saturated fatty acids have been demonstrated to enhance dyslipidemia, while polyunsaturated fatty acids (PUFAs) do the reverse^50^. Taking PUFA omega-3 supplements helps to lower TG levels^51^.

The strength of this study is that it is the first study in Iraq to examine the role of non-HDL and atherogenic indices in predicting glycemic control. One limitation is the lack of information on diet and exercise.

## Conclusion

The prevalence of poor glycemic control among type 2 DM patients was high in Basrah, Iraq. Dyslipidemia was associated with poor glycemic control. The results of this study confirm that the total/HDL cholesterol and LDL/HDL cholesterol ratios are risk indicators of glycemic control with greater predictive value than isolated parameters used independently. Although the strength of the association was weak, non-HDL cholesterol was a significant predictor of glycemic control. In this sense, the use of these indices is highly recommended in a clinical setting as biomarkers for predicting glycemic control as they are simple to compute from frequently and commonly measured lipid parameters in primary care, are cost-effective and identify distinct cardiometabolic problems. Further longitudinal studies are recommended to explore the factors associated with poor glycemic control that were not identified in the current study.

### Source of funding

None

### Conflict of interest

None of the authors declares a conflict of interest

## Figures and Tables

**Table 1:** Basic demographic and clinical characteristics of the study population

Variable	Good glycemic control (HbA1c ≤ 7%), n = 51	Poor glycemic control (HbA1c>7%), n = 278	P-value

Female sex, No. (%)	17 (33.3)	152 (54.7)	0.005

Age (y), Mean ± SD	48.5 ± 11.6	51.5 ± 11.7	0.098

BMI (Kg/m^2^), Mean ± SD	26.8 ± 4.8	29.2 ± 4.9	0.027

Smokers, No. (%)	4 (7.8)	34 (12.1)	0.312

Family history of DM, No. (%)	23 (45.1)	116 (41.7)	0.678

Type of treatment, No. (%)			

Diet only	2 (3.9)	6 (2.2)	0.007

Oral HA	31 (60.8)	113 (40.7)	

Insulin	13 (25.5)	63 (22.7)	

Insulin + Oral HA	5 (9.8)	96 (34.4)	

Duration of DM (y)	8.8 ± 6.2	10.4 ± 7.6	0.211

SBP (mmHg), Mean ± SD	130.1 ± 21.2	139.1 ± 26.6	0.041

DBP (mmHg), Mean ± SD	83.3 ± 11.2	87.7 ± 12.1	0.046

FBG, Mean ± SD	132.6 ± 32.9	243.8 ± 78.9	< 0.001


No. = Number, SD = standard deviation, HbA1c = glycosylated hemoglobin, BMI = body mass index, DM = diabetes mellitus, y = years, SBP = systolic blood pressure, DBP = diastolic blood pressure, FBG = fasting blood glucose

**Table 2:** Association between the lipid profile, the atherogenic indices, and glycemic control

Variable	Good glycemic control (HbA1c ≤ 7%), n = 51	Poor glycemic control (HbA1c>7%), n = 278	P-value

TC (mg/dl)	195.2 ± 49.4	230.2 ± 44.9	< 0.001

TG (mg/dl)	181.3 ± 86.0	238.3 ± 93.4	< 0.001

HDL-C (mg/dl)	41.4 ± 9.4	34.2 ± 10.3	< 0.001

LDL-C (mg/dl)	125.9 ± 41.1	149.1 ± 46.8	0.001

VLDL (mg/dl)	39.4 ± 22.4	47.5 ± 18.7	0.006

Non-HDL (mg/dl)	153.2 ± 49.4	196.8 ± 45.8	< 0.001

Non-HDL/HDL ratio	3.94 ± 1.65	6.47 ± 3.03	< 0.001

TC/HDL ratio (CR- I)	4.92 ± 1.64	7.42 ± 2.98	< 0.001

LDL/HDL ratio (CR- II)	3.20 ± 1.41	4.84 ± 2.57	< 0.001

TG/HDL	4.58 ± 2.43	7.87 ± 4.81	< 0.001

Log TG/HDL (AIP)	0.61 ± 0.22	0.83 ± 0.25	< 0.001


TC = total cholesterol, TG = triglycerides, HDL = high density lipoprotein, LDL = low density lipoprotein, VLDL = very low density lipoprotein, CR-I = Castelli risk index-I, CR-II) = Castelli risk index-II, AIP = atherogenic index of plasma

**Table 3:** Area under the curve (AUC) of the lipid profile and the lipid ratios for glycemic control

Variable	AUC	95% CI	Cutoff value	Sensitivity	Specificity	P-value

TC	0.69	0.61–0.77	202	69.8%	57%	< 0.001

TG	0.68	0.60–0.77	172	80.0%	53%	< 0.001

LDL	0.63	0.55–0.74	132	58.0%	51%	0.002

Non-HDL-C	0.73	0.65–0.81	155	82.4%	51%	< 0.001

Non-HDL/HDL	0.78	0.72–0.85	3.78	85.3%	53%	< 0.001

TC/HDL (CRI- I)	0.78	0.72–0.85	4.70	86.0%	53%	< 0.001

LDL/HDL(CRI- II)	0.72	0.65–0.79	3.10	76.0%	53%	< 0.001

TG/HDL	0.76	0.69–0.83	4.46	78.0%	53%	< 0.001

log TG/HDL (AIP)	0.75	0.68–0.82	0.63	79.1%	51%	< 0.001


TC = total cholesterol, TG = triglycerides, HDL = high density lipoprotein, LDL = low density lipoprotein, CRI-I = Castelli risk index-I, CRI-II = Castelli risk index-II, AIP = therogenic index of plasma

**Table 4:** Logistic regression analysis of the lipid profile and atherogenic indices for glycemic control of diabetes mellitus

Parameter	β Coefficient	OR	95% CI for OR		P-value

			Lower	Upper	

TC	0.071	1.05	0.98	1.18	0.130

TG	0.056	1.03	0.90	1.20	0.488

Non-HDL	0.013	1.01	1.00	1.02	0.020

TC/HDL (CRI-I)	1.598	4.94	2.35	10.41	< 0.001

LDL/HDL (CRI-II)	1.534	4.63	1.96	10.98	< 0.001


TC = total cholesterol, TG = triglycerides, HDL = high density lipoprotein, LDL = low density lipoprotein, CRI-I = Castelli risk index-I, CRI-II = Castelli risk index-II

## References

[1] https://diabetesatlas.org/idfawp/resource-files/2021/07/idf_atlas_10th_edition_2021.pdf.

[15] http://www.nhlbi.nih.gov/guidelines/cholesterol/atp4/index.htm.

